# Proline-rich protein from *S. mutans* can perform a competitive mineralization function to enhance bacterial adhesion to teeth

**DOI:** 10.1038/s41598-022-26303-x

**Published:** 2022-12-23

**Authors:** Kun Tian, Chengwei Xiao, Yue Chen, Min Zhou, Jun Guo, Xiaohua Ren, Qin Du

**Affiliations:** 1 Department of Stomatology, Sichuan Provincial People’s Hospital, University of Electronic Science and Technology of China, No. 32#, Section 2, the First Ring Road West, Chengdu, 610072 Sichuan China; 2 Department of Osteology, Sichuan Provincial People’s Hospital, University of Electronic Science and Technology of China, Chengdu, 610072 Sichuan China; 3 School of Stomatology, Southwest Medical University, Luzhou, 646000 China

**Keywords:** Dental caries, Targeted bone remodelling, Cryoelectron microscopy

## Abstract

A proline-rich region was found in *Streptococcus mutans* (*S. mutans*) surface antigen I/II (Ag I/II). The functions of this region were explored to determine its role in the cariogenic abilities of *S. mutans*; specifically, the proline-rich region was compared with human amelogenin. The full-length amelogenin genes were cloned from human (AmH) and surface antigen I/II genes from *S. mutans*. Then, the genes expressed and purified. We analyzed the structure and self-assembly ability of AmH and Ag I/II, compared their capacities to induce mineralization, and assessed the adhesion ability of *S. mutans* to AmH- and Ag I/II-coated tooth slices. AmH formed ordered chains and net frames in the early stage of protein self-assembly, while Ag I/II formed irregular and overlapping structures. AmH induced mineralization possessed a parallel rosary structure, while Ag I/II-induced mineralization is rougher and more irregular. The *S. mutans* adhesion assay indicated that the adhesion ability *S. mutans* on the Ag I/II-induced crystal layer was significantly higher than that on the AmH-induced crystal layer. *S. mutans’* Ag I/II may have evolved to resemble human amelogenin and form a rougher crystal layer on teeth, which play a competitive mineralization role and promotes better bacterial adhesion and colonization. Thus, the cariogenic ability of *S. mutans* Ag I/II is increased.

## Introduction

It is well known that caries involves the alternating evolution of demineralization and remineralization^1^. Once formed, biofilms wrap around the tooth surface and isolate the oral microenvironment^2^. In theory, this isolation should eliminate as much remineralization as possible, but numerous observations have shown that hydroxyapatite redeposition occurs in decayed tissues^1,3^. We were intrigued and speculated that the biofilm surface might include a hidden functional fragment that directs the formation of hydroxyapatite.

As a dominant bacteria in biofilms, the ability to adhere to the tooth surface is the most important cariogenic basis for *Streptococcus mutans* (*S.mutans*). The extracellular polysaccharides synthesized by *S. mutans* provides binding sites for bacteria, constitutes the main component of the biofilm matrix, together with extracellular DNA, lipoteichoic acids (LTA), saliva proteins^4^. *S.mutans* can also adhere to and colonizes the enamel and produce acidic products through carbohydrate metabolism. This can decrease pH values to below 5.5, resulting in the demineralization of the enamel hydroxyapatite crystals and proteolytic breakdown^2,5^. Antigen I/II (Ag I/II) protein is a major surface protein that functions in adhesin, attaching *S. mutans* to the saliva-coated tooth enamel surface^6,7^. Ag I/II family molecules are conserved in numerous oral streptococci, such as the invasive pathogens *Streptococcus pyogenes* and *Streptococcus agalactiae*^8,9^. The interaction of Ag I/II with host saliva, matrix proteins and other oral bacteria is closely related to the initial adhesion of *S. mutans* on dental surfaces^10^, plaque formation^11^ and caries development^12^.

The formation of tooth enamel involves multiple biological processes such as gene expression, protein secretion, protein folding and assembly, mineral growth and protein degradation. Amelogenin (Am) is the predominant extracellular enamel matrix protein and plays an important role in enamel formation^13^. A polyproline-rich protein matrix, which manifested as the central domain rich in X–Y-proline repeats of Am, is the scaffold that control hydroxyapatites assembly. Similar proline-rich region occur in a wide variety of functionally significant proteins, including mucins, prolamine storage proteins, pancreatic polypeptide hormones, neuropeptides and prion proteins, which perform as mineral-binding domains and internal molecular spacers during the formation of biological minerals and other biocomposites^14^.

Coincidentally, a proline-rich region was detected in surface protein antigen I/II of *S.mutans*, which is a known target of protective immunity^15,16^. This proline-rich region attracted our attention, and we wanted to determine its function during biofilm formation. In view of the above speculation, we compared the structures, morphologies and functions of amelogenin from humans (AmH) and *S.mutans* surface antigen I/II (Ag I/II) to investigate the potential role of the proline-rich region in *S.mutans*.

## Materials and methods

### Bacterial strains and culture conditions

*S.mutans* UA159, *Escherichia coli* Top 10 and BL-21 were used in this study. *S.mutans* strains were grown statically at 37 °C in Todd-Hewitt broth (THB) or on THB agar plates under aerobic conditions with 5% CO_2_. *E. coli* strains were grown in Luria–Bertani (LB) broth or on LB agar plates. Appropriate antibiotics were used to select the transformants on the media as needed.

### Construction and expression of proteins from humans and *S. mutans* respectively

Because human amelogenin was used for comparison, we obtained a complete dental follicle from a third molar in the early stage of amelogenesis. This molar belonged to a 9-year-old boy who was about to undergo orthodontic treatment and needed preventive tooth extraction. Written informed consent was provided by his legal guardians. The study was approved by the Ethics Committee on Human Research of Sichuan Provincial People’s Hospital (NO.20110308, OCT. 19th, 2011), all methods were carried out in accordance with relevant guidelines and regulations. The ameloblast layer was stripped, mRNA was isolated using TRIzol (Invitrogen, USA) and cDNA was obtained by using an iScript cDNA synthesis kit (Qiagen, USA). AmH was amplified using the primers (TTAGAATTCCATATGCCCCTACCACCTCATCCTGG) and (CCTGGATCCTTAATCCACTTCTTCCCGCTTGGTCTT), Ag I/II was amplified using the primers (GATCAGCTAGCCCCACACCGCCGGTTAAACCAACA) and (GATCACTCGAGAGTTGGTACAGATGGAGGTGTTGGA) from an *S.mutans* colony lysate with *BamH*I&X*holR* and was ligated to the recombinant expression plasmid pET28b. The recombinant plasmids were sequenced using T7 primers to verify the correct amelogenin sequences^17^.

### Expression and purification of target proteins

The clones containing the AmH and Ag I/II genes were selected by colony PCR. The expression of AmH and Ag I/II protein was then induced by 0.1 mmol/L isopropythio-β-D-galactoside (IPTG) (Fluka, Buchs, Switzerland), the cell density was obtained with the concentration of IPTG, and the incubation time and temperature were optimized to promote soluble AmH and Ag I/II expression. Recombinant *pET28b-*AmH and *pET28b-*Ag I/II which carry an optional C-terminal His-Tag sequence were transformed into *E. coli* strain *BL21*, collected and centrifuged at 5000 rpm for 2 min. The cells were resuspended in 1 mL of phosphate buffered saline (PBS), sonicated in an ice bath for 10 min, and centrifuged at 10,000 rpm for 10 min at 4 °C. The induction of protein expression was analyzed via SDS-PAGE followed by Coomassie brilliant blue (CBB) staining. One liter of bacterial culture for each induced recombinant strain was pelleted via centrifugation (12,000 rpm for 30 min at 4 °C), resuspended in 30 mL lysis buffer (20 mM Tris pH 8.0, 500 mM NaCl) and lysed via sonication (10 min, repeated 5 s on, 10 s off cycle). Cell debris was pelleted by centrifugation for 60 min. The AmH and Ag I/II in the supernatant were then purified with a HisTrap column (Ni-affinity column) for 30 min. The column was washed with binding buffer, and the AmH and Ag I/II proteins were eluted by an elution buffers containing a linear gradient of imidazole ranging from 50 to 300 mM. The eluted proteins were then centrifuged at 3600 rpm for 30 min at 4 °C, filtered with a 0.22 μm hollow fiber membrane, and loaded onto an anion-exchange Resource Q packed column (GE Healthcare, Piscataway, USA) which was preequilibrated with Buffer A (50 mM NaCl, 25 mM Tris–HCl pH 8.0). The columns were then washed with Buffer A, and the AmH and Ag I/II proteins were eluted with a linear gradient of 100% buffer A to 100% buffer B (1 M NaCl, 25 mM Tris–HCl pH 8.0). The proteins were further purified using a 16/60 Superdex 75 gel-filtration column (GE Healthcare) to near homogeneity and were maintained in a solution containing 20 mM Tris–HCl (pH 8.0) and 100 mM NaCl. Proteins were analyzed via liquid chromatography-tandem mass spectrometry (LC/MS/MS), lyophilized and stored at − 20 °C.

### Self-assembly assay

Protein stock solutions were prepared by dissolving of the lyophilized recombinant amelogenin in distilled water at a concentration of 10 mg/mL. The solution was adjusted to pH 3.5 (the monomer form was maintained without polymerization^18^)using HCl and was stored^19^, for at least 24 h at 4 °C prior to the experiments to ensure that the protein completely dissolved.

For cryo-electron microscopy (EM), the 1-, 10- and 20-min assembly reactions were set up by further diluting the protein stock 10 times with 4 mM phosphate-buffered saline (PBS, pH 7.4). Three microliters of the reaction was applied to glow-discharged 200 mesh Quantifoil R 2/1 grids (Electron Microscopy Sciences, Hatfield, PA), blotted briefly and plunged into liquid ethane. The frozen grids were transferred to a Gatan 622 cryo-holder and observed in an FEI Tecnai F20 electron microscope (Eindhoven, Netherlands) operated at 200 kV with magnifications from 5,000 × to 80,000 × and defocus settings of − 2.0 to − 5.0 μm. Images were collected under low-dose conditions on a Gatan Ultrascan 4000 CCD camera.

### In vitro induction of crystal growth

In vitro inductions of crystal growth were tested on glass slices and demineralized human tooth slices. The slices were divided into 16 groups with 2 samples in each group, for a total of 32 samples. The specific grouping is shown in Table S1. The tooth slices were prepared from human third molars without fillings (the teeth were extracted following the standard procedures for extraction at Sichuan Medical Science Academy and Sichuan Provincial People's Hospital). The teeth were treated with 3% sodium hypochlorite for 1 min to remove bacteria and were rinsed with PBS. Slices of 0.2–0.5 mm thickness and 10 mm diameter were cut longitudinally or transversely with a low-speed diamond saw cooled by water. The demineralization was performed by using 30% phosphoric acid (H3PO4) for 30 s. A 1 mg/mL protein solution (AmH, Ag I/II, AmH + Ag I/II 1:1(v/v), pH 3.5) was brushed onto the tooth and glass slices and air-dried for 10 min. Crystallization was performed by immersing the slices in the biomimetic calcification solution with 2.58 mM calcium (CaCl2·2H2O, > 74.4%CaCl_2_) and 1.55 mM phosphates (KH_2_PO_4_, > 99%) at 37 °C, in 50 mM pH 7.6 Tris–HCl and 180 mM NaCl buffer. The calcification solution pH was adjusted to 6.7 with 1 M HCl using a Metrohm 718 pH–STAT. After a designated time (1 day, 7 days), the tooth slices were removed from the solution, rinsed with running deionized water for 50 s, air-dried and studied under a scanning electron microscope (SEM). Then, SEM images were acquired with a Quanta FEG 650 under a high vacuum. Prior to imaging, the samples were prepared with an Au–Pd sputter coating to promote surface conductivity and reduce charging artifacts. An accelerating voltage of 15 kV was used for imaging.

### Bacteria adhesion assay

Overnight, cultures of *S. mutans* were recultured into fresh THB and grown to an OD600 of 0.6. The culture was diluted to 1:100 with THB and subsequently aliquoted into 14 mL FALCON tubes and incubated with experimental tooth slices (normal tooth, demineralized tooth, remineralized tooth, AmH, Ag I/II and AmH + Ag I/II groups) for 16 h at 5% CO_2_ at 37 °C under static conditions. After an overnight culture, the tooth slices were washed three times using PBS and stained with 2.5 µM Syto-9 for 30 min at room temperature. The samples were examined with a Nikon Elipse 90i microscope equipped with an epifluoresence and NIS elements AR imaging system. Images were captured as instructed by the manufacturer. Fluorescence intensity was quantified by the method of Gassmann et al*.* using ImageJ 1.48v (Windows Version of NIH Image, http://rsb.info.nih.gov/nih-image/).

### Statistical analysis

The data were analyzed via SPSS 24.0 (SPSS Inc, Chicago, 236 IL, USA). Student’s t test was used to determine the statistical significance of these indices. P values < 0.05 were considered to be statistically significant.

## Results

### Characteristics of recombinant proteins

**Figure 1:**
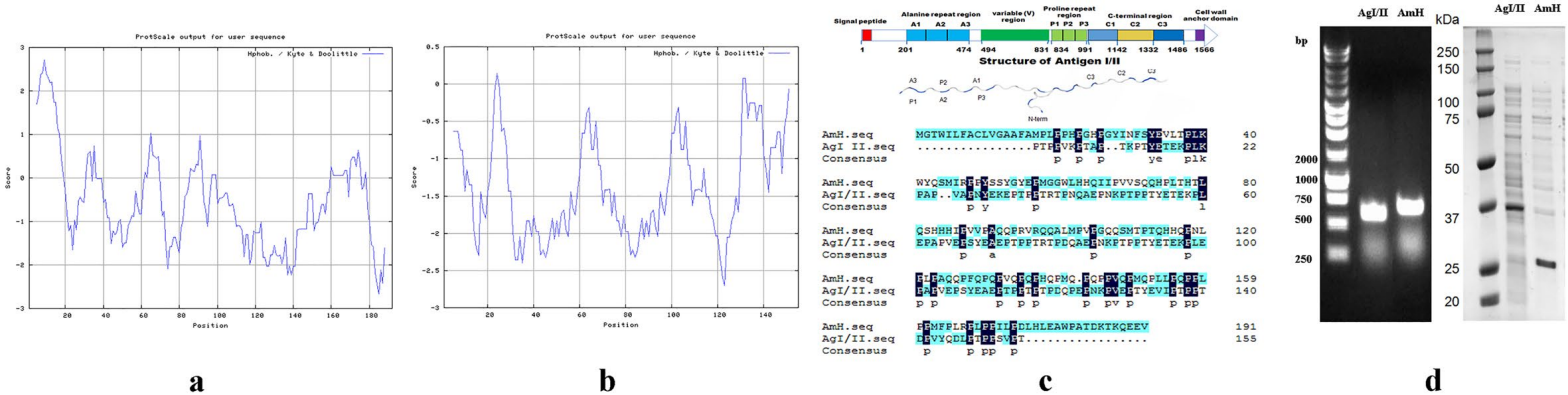
Characteristics of amelogenins and Ag I/II. (**a**) Hydrophobicity plot of AmH; (**b**) Hydrophobicity plot of Ag I/II; (**c**) Schematic representations of Ag I/II primary and tertiary structures. Position of Ag I/II and sequence comparison between AmH (upper) and Ag I/II (down). Ag I/II of *S.mutans* contains 1,566 amino acids (AA): with an N-terminal signal motif, followed by an alanine repeat region (201aa to 474aa) and a proline repeat region (834aa to 991aa). The cell wall anchor domain is located at the C-terminus. AmH and Ag I/II share 19.4% identity and 24% similarity of protein sequence. Most of identical amino acids are proline; (**d**) Agarose gel electrophoresis (1%) of the colony PCR products showing the BL21 clones containing AmH and Ag I/II gene. M, DM2000 Marker; SDS gelatin electrophoresis showed that the molecular weight of AmH about 27 kd, Ag I/II about 34 kd.

Based on the full-length sequence of *S. mutans* UA159 published by GenBank (NCBI NC_004350.2), the functional fragment was defined by the reference protein sequence analysis network server, and the proline repeat sequence of surface protein antigen Ag I/II (1562AA) was identified as the working fragment (Fig. 1), which is the functional extract of amyloid protein. The protein hydrophobicity scale was analyzed with the Protscale tool at ExPASy (https://web.expasy.org/protscale/). AmH has a hydrophilic C-terminal domain and a hydrophobic N-terminal domain (Fig. 1a). Most domains of Ag I/II (Fig. 1b) were hydrophilic because the main domain was under the standard line.

Ag I/II from *S. mutans* contains 1,566 amino acids (AA), beginning with an amino-terminal signal motif, followed by an alanine repeat region (202aa to 474aa), a proline repeat region (836aa to 991aa) and a cell wall anchor domain located on the C-terminus (Fig. 1c). The alanine repeat region is comprised of three complete alanine-rich repeats (82aa residues each) with ∼ 30% alanine content. The proline repeat region contains three proline-rich repeats (39 residues each) with ∼ 35% proline content. Compared to AmH, Ag I/II shares 19.4% identity and 24% similarity in terms of protein sequence. Most of the identical amino acids are proline.

The AmH and antigen I/II were successfully cloned that confirmed by direct sequencing expressed and purified (Fig. 1d). After transfection, identification, purification get protein liquid extract, SDS gelatin electrophoresis showed that the molecular weight of AmH about 27 kd, in line with the calculated molecular weight (Fig. 1d). Ag I/II via https://model.nmr.ru/preddimer/ predicts that in the solution to form dimers, molecular weight and protein electrophoresis showed the molecular weight is calculated using (17kd) twice about 34kd.

### Ag I/II formed more irregular clutters than AmH

**Figure 2:**
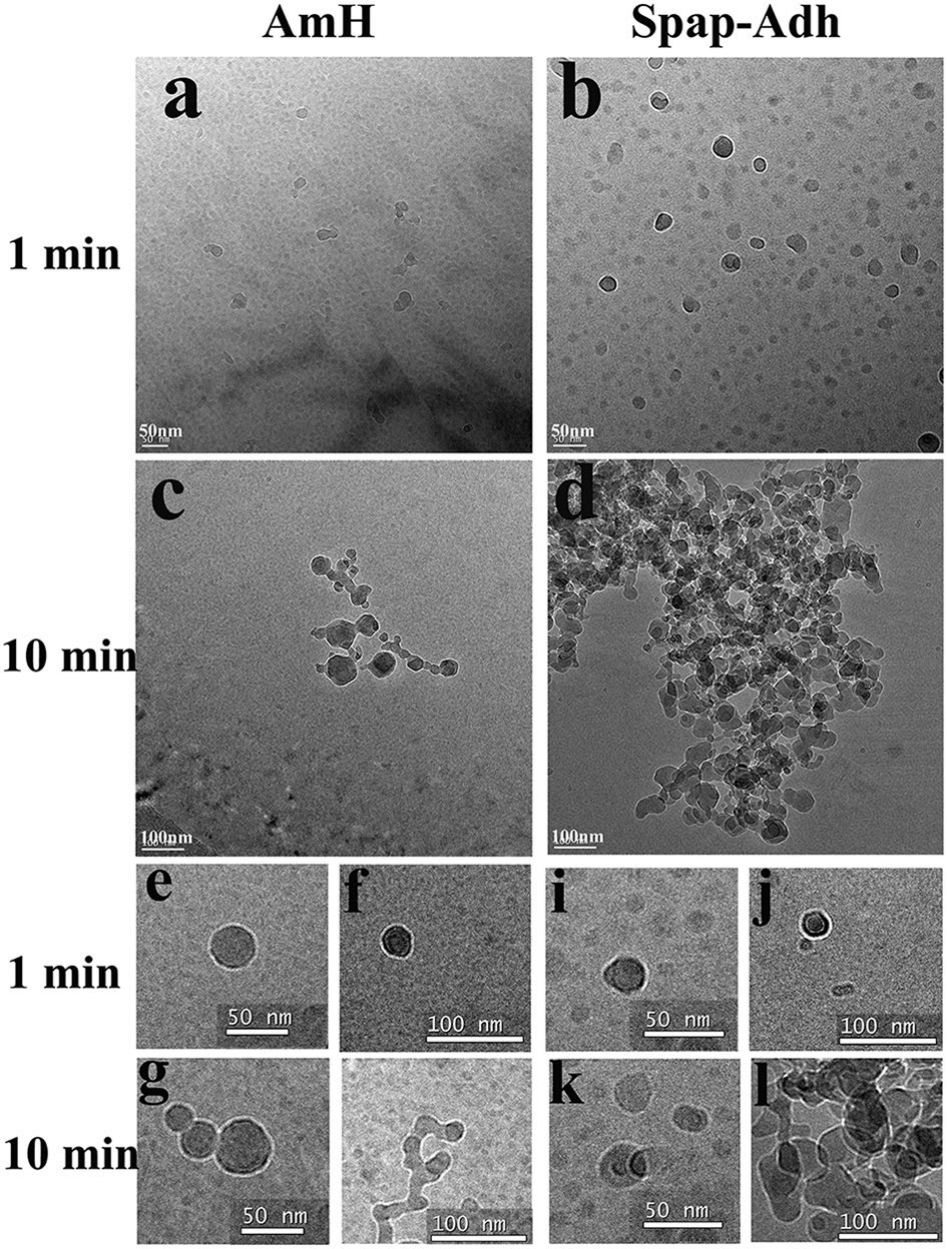
Cryo-TEM micrographs of AmH and Ag I/II; (**a**) AmH at 10 min, full of monmer, dimer and oligomer. (**b**) Ag I/II at 10 min, the tiny particles larger than AmH; (**c**) AmH at 20 min, quaternary structures were legible (**d**) Ag I/II at 20 min, bushy nest clearly visible; (**e**–**h**) amplified of quaternary structures in AmH, (**e**, **f**) nanospheres; (**g**) nanochains, the way to build nanochains were end-to-end junction (**h**) net structure, distinct and scattered; (**i**, **l**) amplified quaternary structures in Ag I/II, (**i**, **j**) nanospheres; (**k**) nanochains; (**l**) cluster structure, stacked up into piles.

The structures of the assembled AmH and Ag I/II were observed under TEM after a 1-min incubation in PBS (pH 8.0). Both AmH and Ag I/II existed as 3–5 nm particles, which was consistent with previous observation of amelogenin monomers. After 10 min of incubation, dimers and oligomers (Fig. 2e, i) emerged with larger ring-like structures. The AmH ring included six to eight monomers and had a diameter of 5–10 nm (Fig. 2a), as compared to the Ag I/II ring, with six to ten monomers and a diameter of 10–15 nm (Fig. 2b). The larger irregular spherical particles, with an average diameter of 10–40 nm, were identified as amelogenin nanospheres (Fig. 2f,j). After 20 min of incubation, more highly ordered constructions were observed. However, there were obvious differences between the AmH and Ag I/II structures. The oligomers and nanospheres of AmH formed nanochains and reticulate structures (Fig. 2c). On the other hand, the oligomers and nanospheres of AgI/II dispersed to cluster with a stronger density (Fig. 2d). Interestingly, individual multistep processes were found in both AmH and Ag I/II. The AmH oligomers and nanospheres formed nanochains in an end-to-end highly connected pattern (Fig. 2g,h). The Ag I/II oligomers and nanospheres overlapped repeatedly, forming more irregular clusters (Fig. 2k,l). The results suggested that as compared with AmH, which was able to construct ordered chains and net frames, Ag I/II constructed more irregular and overlapping clutters.

### Ag I/II-induced crystals are different in shape from AmH-induced crystals

**Figure 3:**
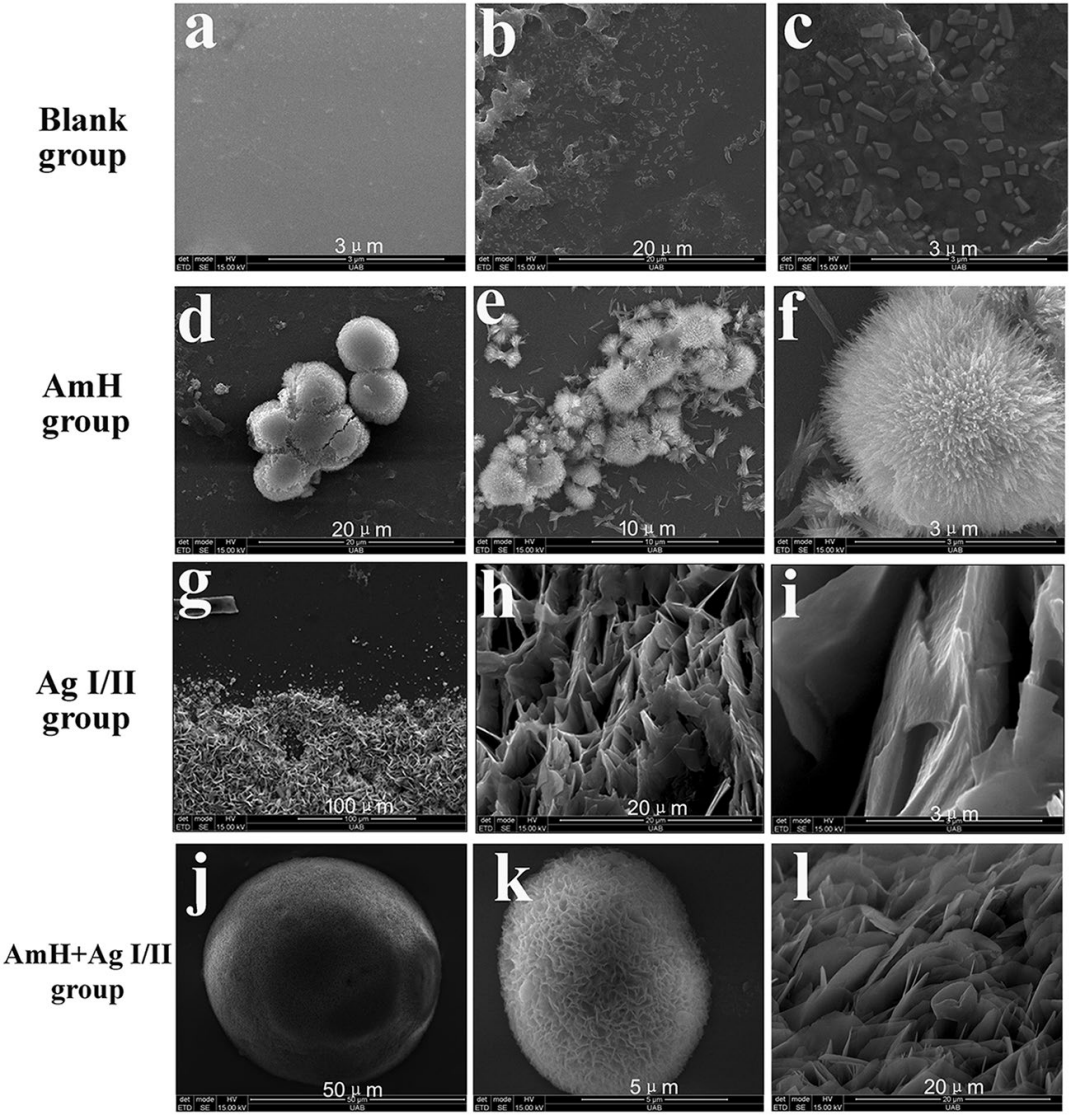
SEM images of newly grown crystals on the glass slide after mineralization. (**a**–**c**) blank group, (**a**) smooth glass slide; (**b**, **c**) amorphous deposits in triangular, tetragonal or polygonal shapes. (**d**–**f**) AmH group, (**d**) globular crystals in 3–5 μm diameters, scatted in knots; (**e**) between the globules, needles/rods were bound together in bundles; (**f**) thousands of needles/rods grow from the core and looks like a hedgehog. (**g**–**i**) Ag I/II group, (**g**) dense flake crystals cover the surface; (**h**) flake crystals standing at random; (**i**) flake crystals with irregular shape. (**j**–**k**) AmH + Ag I/II group, (**j**) isolated globular crystals, with diameter from 5 to 40 μm; (**k**) petal-like crystals aggregate into globule; (**l**) petal like-crystals.

**Figure 4:**
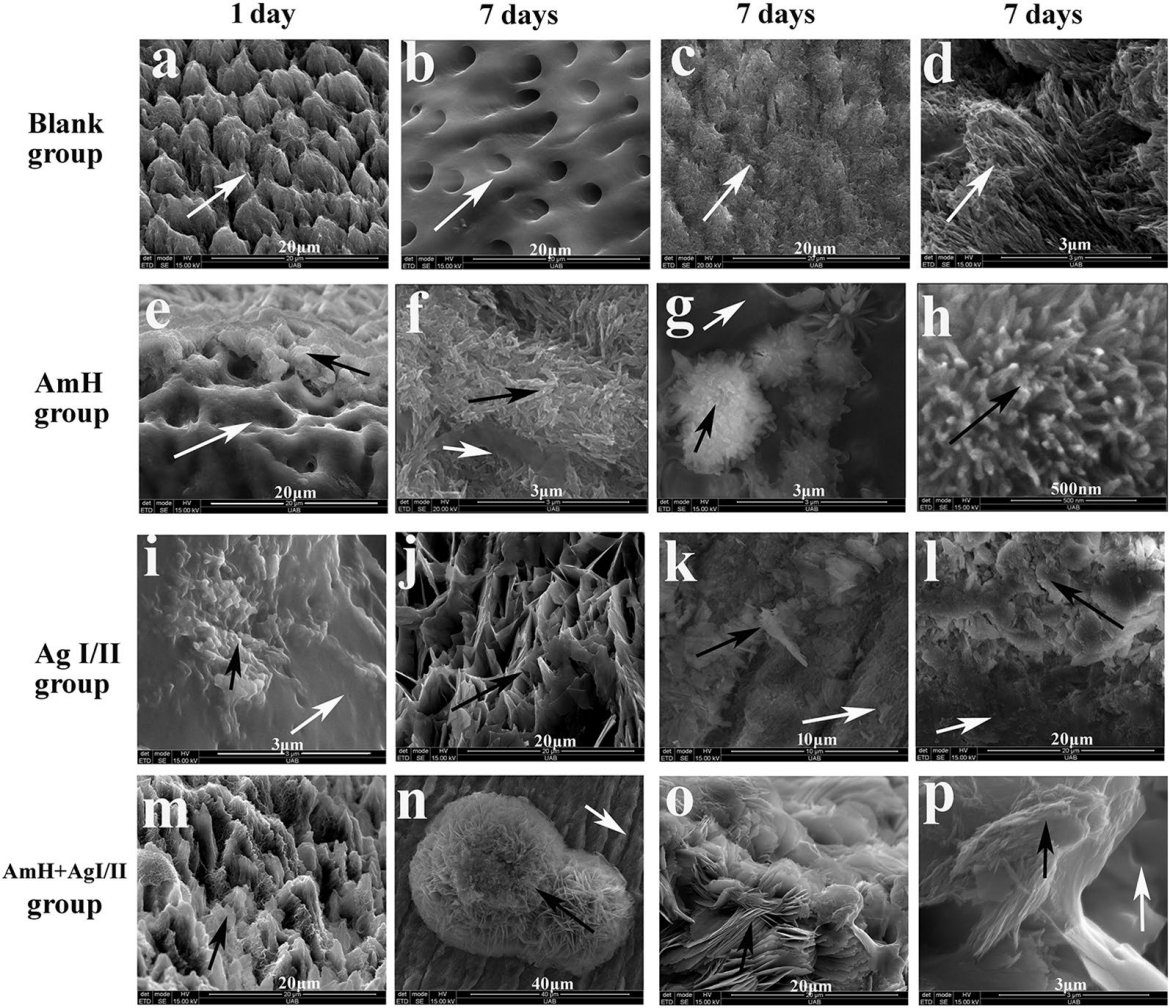
SEM images of newly grown crystals (black arrows) on the tooth structure (white arrows) after mineralization. (**e**, **i**, **m**) are the early stage of mineralization (1 day), the other reactions continued 7 days. (**a**–**d**) normal tooth, (**a**) demineralized enamel rod; (**b**) demineralized dentin tube; (**c**) demineralized rod and inter-rod woven; (**d**) bundle crystals in enamel rod at high magnification. (**e**–**h**) AmH group, (**e**, **f**) 0.5–1.2 μm thick new layer attached to enamel rod and dentin tube; (**g**) hedgehog crystals globule; (**h**) radial needles/rods which look similar to bundle crystals in normal enamel rod. (**i–l**) Ag I/II group, 1–10 μm thick new layer deposited on surface of tooth (**i**) polygonal; (**j**) fractured sheet over 8 μm; (**k**) irregular block; (**l**) tiny stumps. (**m**–**p**) AmH + Ag I/II group, petal-like crystals with size of 2–20 μm, (**m**) prototype of sheet shape; (**n**) spherical sheet type same in glass surface; (**o**) petal crystals in sphere; (**p**) parallel needle-like structure attached to the petal.

To observe the crystals morphology induced by Ag I/II and AmH, the surface features of samples were analyzed via scanning electron microscopy. The crystallizations on the glass slices were observed first. In the control group, amorphous deposits can still be observed without any protein guidance (Fig. 3a–c). The deposits were 15–50 nm in diameter and were of triangular, tetragonal or polygonal shapes. In the AmH group, a globular crystals of 3–5 μm diameter scattered in knots, bundles of crystals scattered around the globule and needle/rod-like crystals were observed to grow from the core, which made the globule look like hedgehog (Fig. 4d) under higher magnification. The crystal bundles were organized as an aggregation of long needle/rod-like crystals (Fig. 3e,f). It seemed that the crystals bundles were in the early stages of the crystal globule development process. Apparently, the structure and shape of Ag I/II -induced nascent crystal was remarkably different, as shown in Fig. 3g,h. Dense flake crystals covered the glass randomly, and all crystals were irregular in shape. It’s very interesting that in the AmH + Ag I/II group, the nascent crystals were arranged in a 5–40 nm diameter (Fig. 3j,k) distinctive globular shape, which was composed of sub-units with petal-like crystals (Fig. 3l). With the mixture of AmH and Ag I/II, the crystallization structure had mixed characteristics: a sphere-like structure similar to that of AmH crystallization but with a flake/pedal like component similar to that observed in Ag I/II crystals.

Crystallization (black arrow) differences between AmH and Ag I/II were also observed on tooth slices because the tooth surface (white arrow) has unique morphological characteristics such as inner dentin tubes and inter-enamel rods (Fig. 4a–d). In the very beginning stage (1st day) of crystallization in the AmH group, gathered block and long needle/rod-like crystals developed from the enamel rod and inter-rod (Fig. 4e,f), which had a thickness of 0.5–1.2 μm and overlapped most of the exposed surface. Hedgehog-like globules were also observed (Fig. 4g), and the long needle/rod-like crystals with the parallel array resembled enamel rod crystals (Fig. 4h). However, the crystals induced by Ag I/II presented highly disorganized and irregular shapes (Fig. 4i–l). In the mixed group of AmH + Ag I/II, the nascent crystals combined the characteristics of a radical needle/rod (AmH) and scattered sheet (Ag I/II), showing a petal-like globule. Interestingly, some parallel needles/rods grew on the petal sheet (Fig. 4m–p), which suggested that the mineralization induction capacity of AmH is stronger than that of Ag I/II. The typical AmH characteristics, such as the spherical structure and the rod details, were well represented.

### Increased adhesion of *S. mutans* to AmH- and Ag I/II-coated tooth slices

**Figure 5:**
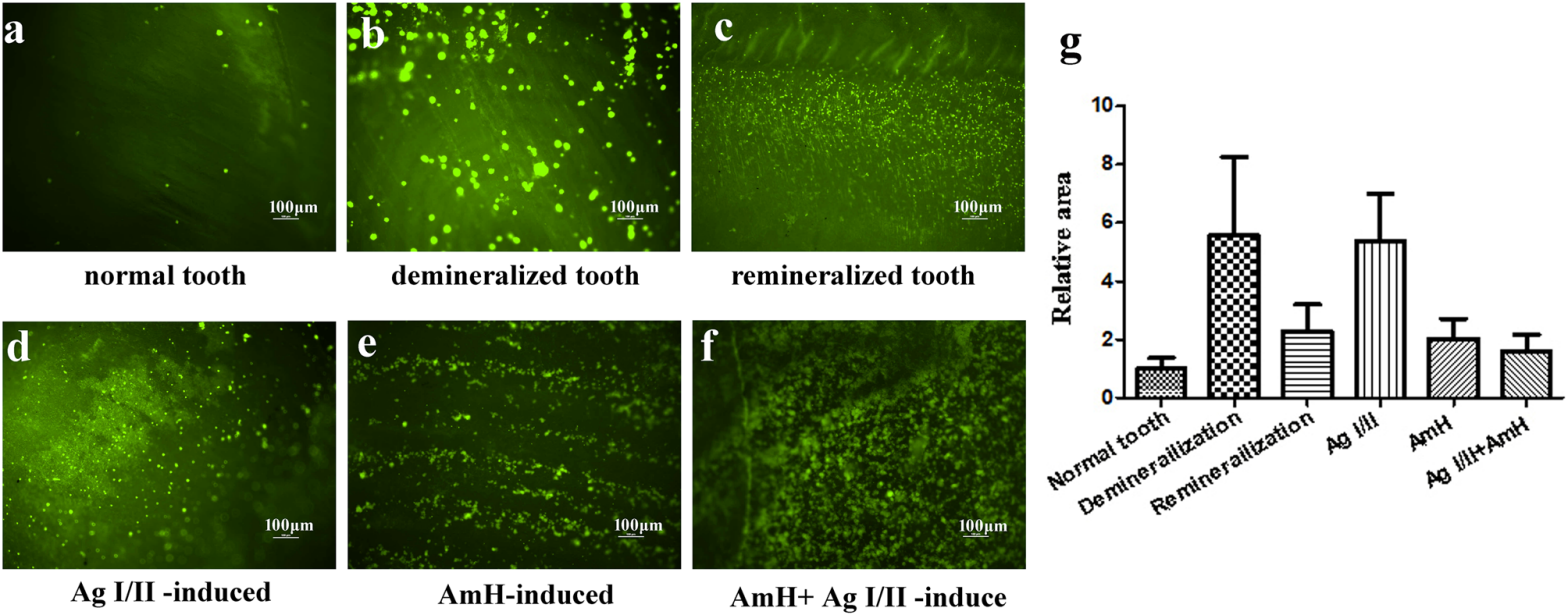
Fluorescence microscope images of *S. mutans* adhesion on the treated surface of the tooth. (**a**) normal tooth, (**b**) demineralized tooth, (**c**) remineralized tooth, (**d**) Ag I/II-induced crystals, (**e**) AmH-induced crystals, (**f**) AmH + Ag I/II-induced crystals. The right chart is the value of relative fluorescence intensity in each group. The demineralized group has the coarsest surface, and AmH group ranked second in this regard. The value for relative fluorescence intensity in the AmH + Ag I/II group was close to that if the remineralization group and higher than that of the AmH group. A Student’s t-test showed that the differences between the normal tooth vs demineralization (P = 0.0414), normal tooth vs Ag I/II (P = 0.0053) Ag I/II vs remineralization (P = 0.0064) were statistically significant.

**Figure 6:**
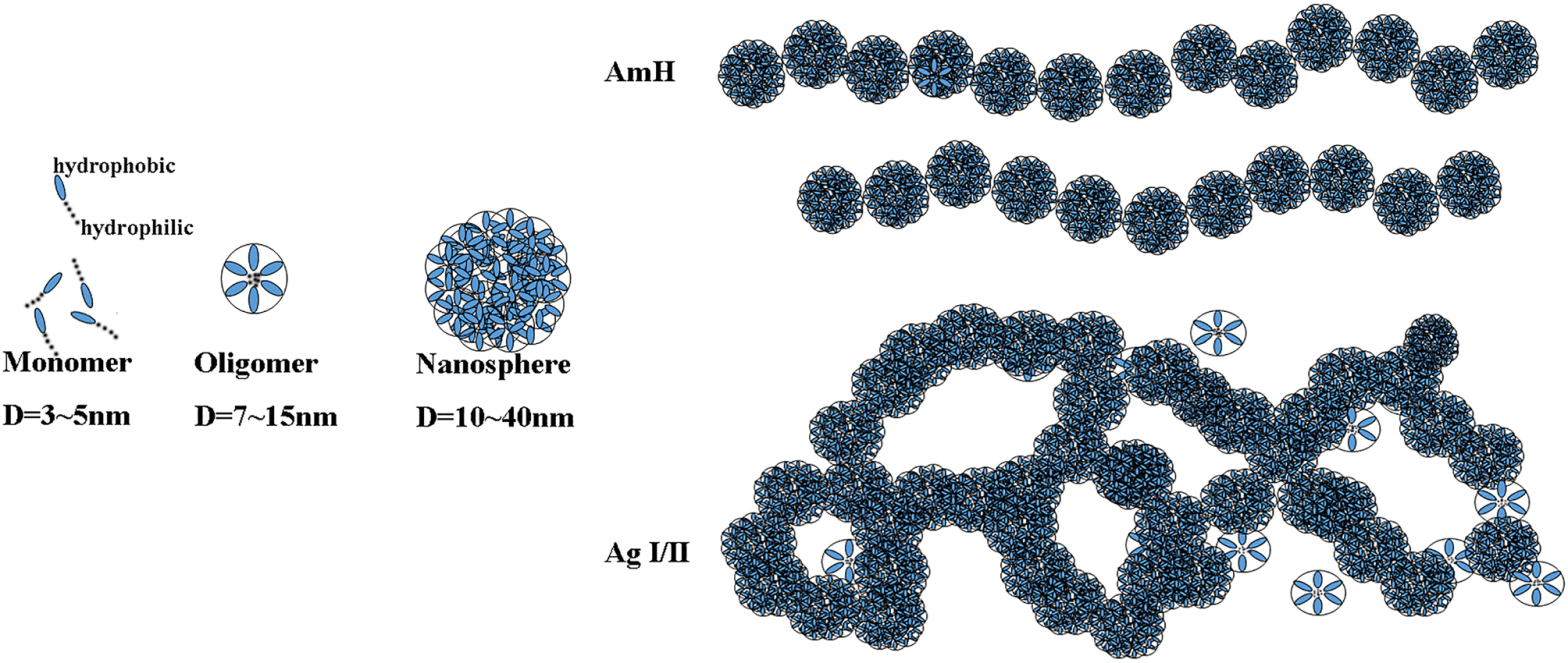
Schematic model of nanochains and nanosphere net. AmH showed end-to-end connection; Ag I/II showed body-to-body connection. The monomers (D = 3 ~ 5 nm), oligomers (D = 7 ~ 15 nm) and nanospheres (D = 10–40 nm) in AmH and Ag I/II were probably comparable in size. However, the long linear structure of Ag I/II offered open-ended connection sites, which induced body-to-body link. As compared to the end-to-end connections seen in AmH, this multipoint joint increased the number of connection sites and promoted multiple types of crystal nucleation and growth. Therefore, the final structures of AmH and Ag I/II would be nanochains and a nanosphere net.

*S.mutans* adhesion assays were performed on normal, demineralized and remineralized teeth with crystal layers induced by Ag I/II, AmH and Ag I/II + AmH. As shown in Fig. 6, the bright spots were *S.mutans* attaching on the tooth surfaces. *S. mutans* showed the highest adhesion ability in the demineralization group. The Ag I/II group was the second most ideal surface for bacterial adhesion, with the mass crystal coverage on the tooth suggesting that it served as an ideal surface for cell adhesion. The adhesion abilities of *S.mutans* for normal, remineralized, AmH and AmH + Ag I/II teeth were significantly lower than those for demineralized tooth and the Ag I/II group (Fig. 5), which indicated that Ag I/II generated an adherent crystal surface to increase bacteria adhesion.

## Discussion

The matrix of mature enamel is composed of hydroxyapatite (HAP) and fluor-hydroxyapatite (FHAp), forming highly ordered structures. The fundamental units of enamel are the rod and interrod, which are woven from parallel crystal bundles. This unique construction is believed to result from the orchestrated receding of the ameloblast cell layer, as each rod has approximately the same diameter as a single cell^20^. Ameloblasts express and secrete proteins, together with proteases, to form an extracellular matrix that controls enamel development. Amelogenin (Am), which is the most abundant component (90%) among all enamel structural proteins, exhibits a capacity of self-assembly to form supramolecular nanospheres and causes parallel crystal bundles to twist into enamel rods^21^. It is well established that Am shows the potential to control calcium phosphate polymorphism and modulate crystal morphology both in vivo^22,23^ and in vitro^24,25^.

Amelogenin comprises the following domains: a hydrophobic tyrosine-rich N-terminal domain (TRAP)^22^, a charged C-terminal hydrophilic telopeptide (C telopetide) and a central domain rich in X–Y-proline repeats^26^. It appesrs that these primary structural motifs are highly conserved and have remained unchanged for 250 million years^27^. When the pH, temperature and concentration change, the amelogenin monomers selfassembled into quaternary structures, including nanospheres, oligomers^28^, nanochains and microribbons^29^, through intermolecular hydrophobic interactions while the hydrophilic segment of each molecule is exposed on the nanosphere surface. Recent studies have shown that various biomimetic systems use Am^30,31^, the functional fragment of Am^32^, amelogenin-containing hydroge^33^, and amelogenin analog^34,35^ to repair enamel defects.

Although the spatial structure of amelogenin is well characterized, the potential array mode of the Am molecule, which is obtained through its amphiphilic sequence property, remains uncertain. Full-length recombinant amelogenin can self-assemble into oligomers and nanospheres in various solvents^18^. As in previous reports, after a 20-min reaction at pH 8.0 PBS, both the oligomers and nanospheres of AmH and Ag I/II in our study formed nanochains and reticulate structures. However, the diameters of both AmH and AgI/II nanospheres (10–40 nm) in our study (Fig. 2e,f,i,j) were larger than the previously reported size of 10–20 nm.

In our study, the structures of the AmH and Ag I/II monomers resembled one another, but the connection modes between nanospheres were completely different. AmH showed an end-to-end joint pattern, while Ag I/II exhibited an overlapping chain pattern. Due to the end-to-end joint of the nanospheres in AmH nanochains were the main body, and the overlap chains of Ag I/II fit together into a network.

The porcine Am rP172 monomer was found to exhibit a globally unfolded state^36^, and the presence of a local residual secondary structure [α-helix, extended β-strand, turn/loop and polyproline type II (PPII)] may play several functional roles. In our study, we propose that this globally unfolded state may be determined by its amphiphilic property. More specifically, oligomers and nanospheres assemble with intermolecular contacts. In the case of AmH, the end-to-end connection was the only joint molecule–molecule interaction, resulting in chain-like assembly. Matthew R. Larson and others have solved the *S. mutans* Ag I/II structure from A3 to P1. In the structure of Ag I/II, A3 is an extended α-helix (110 residues), and P1 residues (834–874) fold over to interact with A3. In a structural model of P1, the globular head domain that intervenes in the alanine- and proline- repeats away from the cell surface at the tip of a long and narrow extended stalk with the N-terminal region in close proximity to the C-terminal region (Fig. 1c). The A-repeats form a long α-helix that intimately intertwines into a left-handed supercoiled structure with the helical polyproline P-repeats to form the stalk^37^. Furthermore, the long linear structure provided an open-ended connection site, which allowed body-to-body links instead of the end-to-end connections observed in the AmH-treated group. This multipoint joint property increased the number of connection sites and promoted multiple modes of crystallization, nucleation and growth (Fig. 6).

Given the above results, it was not surprising that triangular, tetragonal or polygonal patch crystals (Fig. 3c) in Ag I/II, which matched the nanochains and needle-like crystals (Fig. 3f) in AmH that matched the nanospheres. Unlike the complex structure of teeth, the surface of glass is smooth, which is an ideal place to observe the nucleation and growth of new crystals. There is no significant time division between the protein self-assembly and the mineralized nuclear reactions that occur on the solid surface. Here the mineralized fluid acts as a double function of raising pH to initiate self-assembly and providing calcium and phosphorus ions to achieve mineralization. The classical theory of crystallization in amelogenesis postulates that proteins shape crystallites by specifically inhibiting ion deposition in the crystal sides, orient them by binding multiple crystallites and establish higher levels of crystal organization^35^. However, this theory dose not explain how AmH induced the growth of needle-like crystals on the glass surface. Specifically, a crystal ribbon bond in the central section of several needle-like prisms (Fig. 3) formed the core of the final sea urchin-like crystal bundle (Fig. 3f). The most interesting crystal morphology was seen in the AmH + Ag I/II group. The AmH group lost the needle-like crystal type, and the flat distribution disappeared in the Ag I/II group. The newly formed structure was a crystal globe composed of sheet crystals.

**Figure 7:**
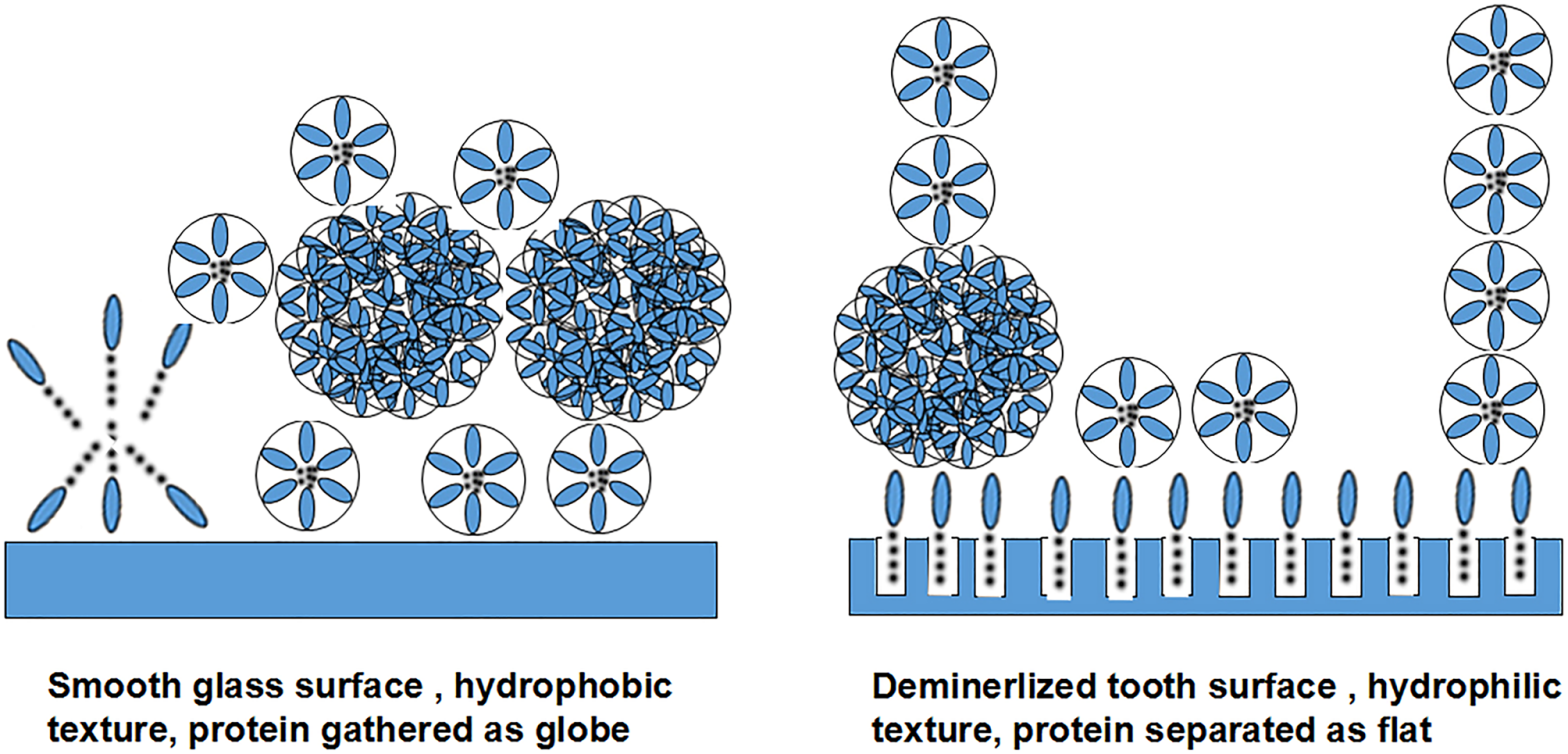
Suggested mechanism for crystal growth on normal glass and demineralized tooth. The smooth and hydrophobic surface of glass reduces interfacial energy and forms a single globe to “stand on”. The demineralized tooth surface presents hydrophilic features and deep groove, which allowed the insertion of the hydrophilic head of Am, leaving the hydrophobic domain to stick out.

Although the crystallization shape was bizarre, it confirmed our deduction that AmH induced stick crystals and developed into balls on the base of the nanochain structure. AgI/II promoted sheet and polygonal block crystals to form a nanosphere net. The mixed AmH/AgI/II produced petal crystals into a globe because of its hybrid nanochain net. A suggested mechanism for how the nanospheres attached to the surface of the matrix is shown in Fig. 7. The texture of the glass surface is smooth and hydrophobic; thus, Am had to create assemblies to reduce the interfacial energy and form a single globe to “stand on” the surface of glass (Fig. 3). In contrast, the demineralized tooth surface is hydrophilic and uneven due to the dissolution of hard tissue. The hydrophilic head of Am was deeply inserted to the uneven groove; the hydrophobic end protruded into the surface and formed a new layer on top of the surface (Fig. 7). We copied and expressed the full length of Ag I/II, but the only Ag I/II that guided the crystallization of hydroxyapatite was P1P2P3. AmH also contained this segment, and the crystals guided by this core segment were messy. The hydrophilic C-terminal and hydrophobic N-terminal in AmH perhaps determine the direction and diameter of crystal growth.

It has been further suggested that mineral morphology and organization in amelogenesis are determined prior to crystallization^38,39^. In our study, the initial stage of apatite growth, as shown in Fig. 4e,i,m, strongly supported this point. A short column of new crystals developed from the AmH-induced layer (Fig. 4e), an irregular slice from Ag I/II (Fig. 4i) and a uniform sheet from the AmH + Ag I/II group (Fig. 4m). Collagen is the major organic component on the surface of demineralized dentin, which constitutes 70% of organic matter^40^. However, after decalcification, the exposed collagen was unwoven and disordered compared to the collagen embedded in dentin, so the shapes of the crystals induced by residual collagen were sheet and laminar; thus, the self-repair of caries were reassembled during the stationary phase under physiological conditions. During the process of plaque formation on teeth, the initial adhesion of the “early colonizer” to the surface is a very important step^41^. A clear correlation has been noted between the surface roughness of materials and biofilm adhesion^42,43^. In our study, *S.mutans* showed very high ability to adhere on the Ag I/II-coated tooth, which was comparable to that on the demineralized tooth and significantly higher than that one normal and AmH-coated teeth. This result suggests that Ag I/II formed a rougher surface on the tooth, increasing the attachment of *S. mutans* to the tooth and further increasing the cariogenic ability of *S. mutans*. Mixed AmH + Ag I/II was used to investigate the crystal shape. Complex products, such as a spherical sheet (Fig. 4n) and petal crystal in a sphere (Fig. 4o), which were bound to the parallel column (Fig. 4p), were found via SEM. The molecular mechanism of the crystallization direction remains unknown, but the sheet shape and parallel column are present in the AmH group and Ag I/II group, respectively. The globe sheet was the integration of the sheet and globe structure, and the petal sheet overlaying the parallel column was a simple physical combination.

In conclusion, amelogenins from humans and the cariogenic bacterium *S. mutans* share similar domains. Proline-rich protein provides competing nucleation sites for mineralization that formed a rough crystal layer on the tooth surface to increase its adhesion to the tooth. Through competitive mineralization, acriogenic ability of proline-rich protein increased. This characteristic of *S. mutans* may lead to new ideas regarding its mechanism of cariogenicity. This study may provide a novel understanding of the microbial dynamics and caries-formation process.

## Supplementary Information

Supplementary Information 1.

Supplementary Information 2.

Supplementary Information 3.

Supplementary Information 4.

Supplementary Information 5.

Supplementary Information 6.

## Data Availability

Antigen Ag I/II sequencing data are available from NCBI (NC_004350.2), the amelogenin sequencing data are available from NCBI (Gene ID:256). The data available upon a reasonable request to the corresponding author.
