# Supplementary material for: Proline-rich protein from S. mutans can perform a competitive mineralization function to enhance bacterial adhesion to teeth
Source: Sci Rep. 2022 Dec 23;12:22250. doi: 10.1038/s41598-022-26303-x (PMC9789152; doi:10.1038/s41598-022-26303-x)
Supplement: Supplementary file 1 — Supplementary Information 1. [file 41598_2022_26303_MOESM1_ESM.docx]

**Supplementary figure legends**

Figure S1: Uncropped image of Fig 1d

Agarose gel electrophoresis (1%) of the colony PCR products showing the BL21 (DE3) clones containing antigen I/II and human amelogenin gene.

Figure S2: Uncropped image of Fig 1d

SDS-PAGE analysis showing antigen I/II and human amelogenin purification using an anion-exchange Resource Q packed column.
