# Supplementary material for: Proline-rich protein from S. mutans can perform a competitive mineralization function to enhance bacterial adhesion to teeth
Source: Sci Rep. 2022 Dec 23;12:22250. doi: 10.1038/s41598-022-26303-x (PMC9789152; doi:10.1038/s41598-022-26303-x)
Supplement: Supplementary file 2 — Supplementary Information 2. [file 41598_2022_26303_MOESM2_ESM.docx]

**Supplementary table legend**

**Supplement Table 1**: Grouping conditions of In vitro induction of crystal growth

The first column lists the carrier for protein attachment, which is also the base for crystal growth. The second column shows the different mineralization reaction times. The first row is the group of experimental proteins, including blank control groups, AmH, Ag I/II, and AmH+ Ag I/II 1:1(v/v). There were 16 groups with 2 samples in each group, for a total of 32 samples.
