# Supplementary figures and images for: Proline-rich protein from S. mutans can perform a competitive mineralization function to enhance bacterial adhesion to teeth
Source: Sci Rep. 2022 Dec 23;12:22250. doi: 10.1038/s41598-022-26303-x (PMC9789152; doi:10.1038/s41598-022-26303-x)

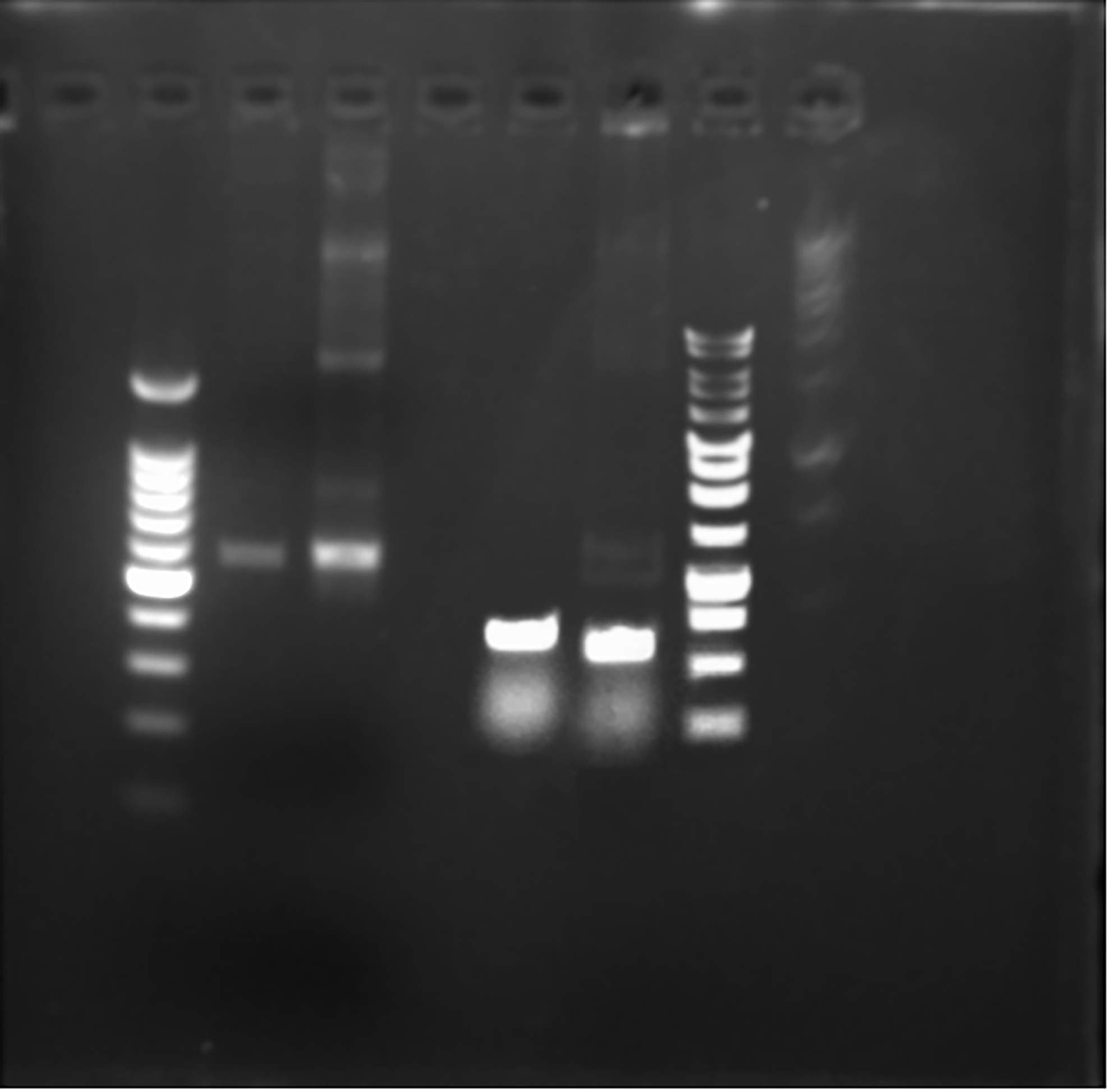

Supplement: Supplementary file 4 — Supplementary Information 4. [file 41598_2022_26303_MOESM4_ESM.tif]

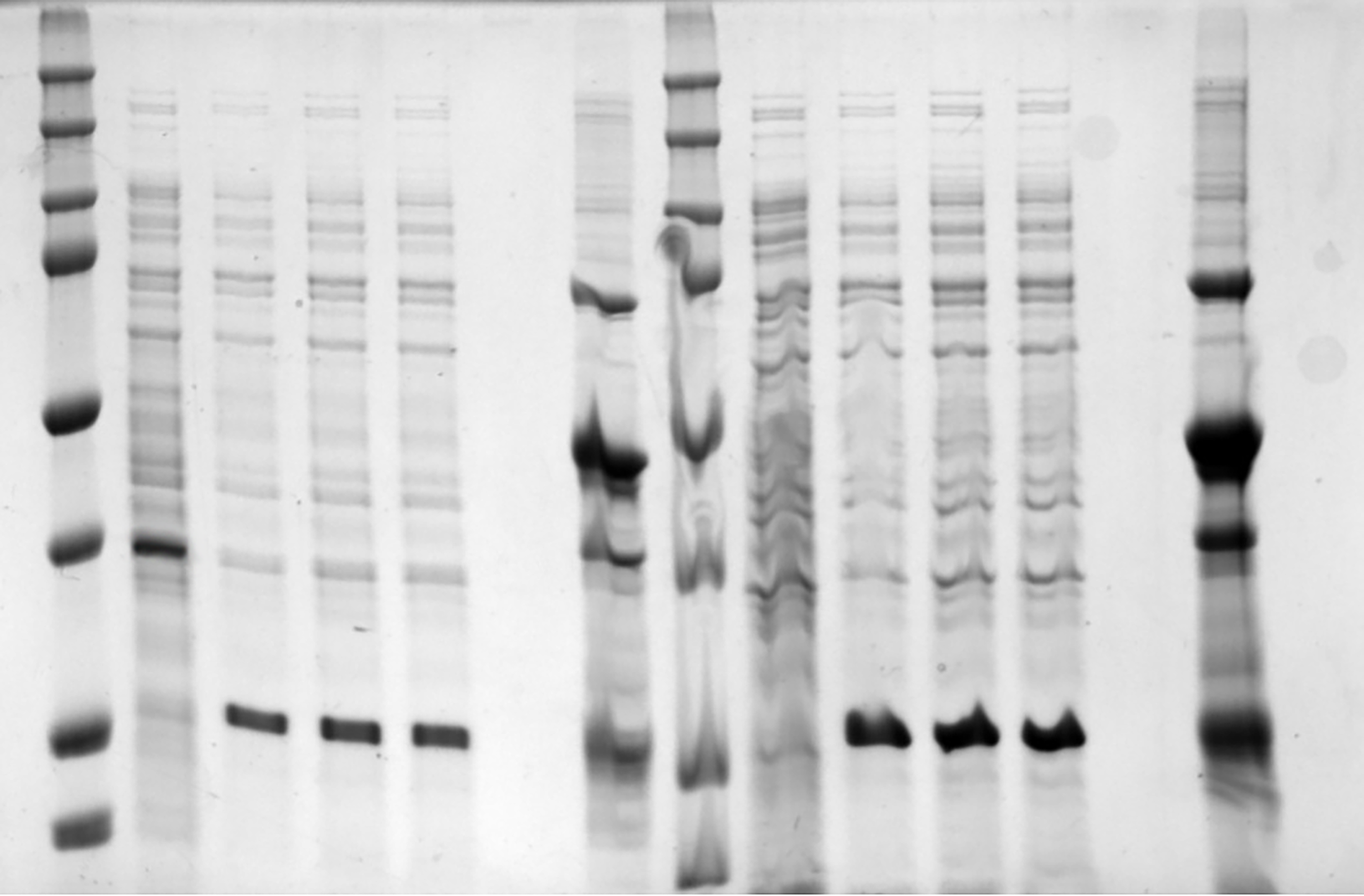

Supplement: Supplementary file 5 — Supplementary Information 5. [file 41598_2022_26303_MOESM5_ESM.tif]
