# Supplementary material for: Proline-rich protein from S. mutans can perform a competitive mineralization function to enhance bacterial adhesion to teeth
Source: Sci Rep. 2022 Dec 23;12:22250. doi: 10.1038/s41598-022-26303-x (PMC9789152; doi:10.1038/s41598-022-26303-x)
Supplement: Supplementary file 6 — Supplementary Information 6. [file 41598_2022_26303_MOESM6_ESM.docx]

Protein sequences of Human Amelogenin

MGTWILFACL VGAAFAMPLP PHPGHPGYIN FSYEVLTPLK WYQSMIRPPY SSYGYEPMGG WLHHQIIPVV SQQHPLTHTL QSHHHIPVVP AQQPRVRQQA LMPVPGQQSM TPTQHHQPNL PLPAQQPFQP QPVQPQPHQP MQPQPPVQPM QPLLPQPPLP PMFPLRPLPP ILPDLHLEAW PATDKTKQEE V

Protein sequences of Antigen I/II

PTPPVKPTAP TKPTYETEKP LKPAPVAPNY EKEPTPPTRT PNQAEPNKPT PPTYETEKPL EPAPVEPSYE AEPTPPTRTP DQAEPNKPTP PTYETEKPLE PAPVEPSYEA EPTPPTPTPD QPEPNKPVEP TYEVIPTPPT DPVYQDLPTP PSVPT
